# Proinflammatory cytokines induce rapid, NO-independent apoptosis, expression of chemotactic mediators and interleukin-32 secretion in human pluripotent stem cell-derived beta cells

**DOI:** 10.1007/s00125-022-05654-0

**Published:** 2022-02-05

**Authors:** Rabea Dettmer, Isabell Niwolik, Karsten Cirksena, Toshiaki Yoshimoto, Yadi Tang, Ilir Mehmeti, Ewa Gurgul-Convey, Ortwin Naujok

**Affiliations:** 1 Institute of Clinical Biochemistry, Hannover Medical School, Hannover, Germany; 2 Department of Digestive and Transplant Surgery, Tokushima University, Tokushima, Japan

**Keywords:** Apoptosis, Cytokines, Differentiation, Immune response, Inflammation, Pancreatic beta cells, Stem cell-derived beta cells, Type 1 diabetes

## Abstract

**Aims/hypothesis:**

The aim of this study was to examine the effects of proinflammatory cytokines on cells of different developmental stages during the generation of stem cell-derived beta cells (SC-beta cells) from human pluripotent stem cells (hPSCs). We wanted to find out to what extent human SC-beta cells are suitable as an experimental cellular model and, with regard to a possible therapeutic use, whether SC-beta cells have a comparable vulnerability to cytokines as bona fide beta cells.

**Methods:**

hPSCs were differentiated towards pancreatic organoids (SC-organoids) using a 3D production protocol. SC-beta cells and non-insulin-producing cells were separated by FACS and differential gene expression profiles of purified human SC-beta cells, progenitor stages and the human beta cell line EndoC-βH1, as a reference, were determined after 24 h incubation with the proinflammatory cytokines IL-1β, TNF-α and IFN-γ via a transcriptome microarray. Furthermore, we investigated apoptosis based on caspase cleavage, the generation of reactive oxygen species and activation of mitogen-activated protein-kinase (MAPK) stress-signalling pathways.

**Results:**

A 24 h exposure of SC-beta cells to proinflammatory cytokines resulted in significant activation of caspase 3/7 and apoptosis via the extrinsic and intrinsic apoptosis signalling pathways. At this time point, SC-beta cells showed a markedly higher sensitivity towards proinflammatory cytokines than non-insulin-producing cells and EndoC-βH1 cells. Furthermore, we were able to demonstrate the generation of reactive oxygen species and rule out the involvement of NO-mediated stress. A transient activation of stress-signalling pathways p38 mitogen-activated protein kinases (p38) and c-Jun N-terminal kinase (JNK) was already observed after 10 min of cytokine exposure. The transcriptome analysis revealed that the cellular response to proinflammatory cytokines increased with the degree of differentiation of the cells. Cytokines induced the expression of multiple inflammatory mediators including *IL-32*, *CXCL9* and *CXCL10* in SC-beta cells and in non-insulin-producing cells.

**Conclusions/interpretation:**

Our results indicate that human SC-beta cells respond to proinflammatory cytokines very similarly to human islets. Due to the fast and fulminant cellular response of SC-beta cells, we conclude that SC-beta cells represent a suitable model for diabetes research. In light of the immaturity of SC-beta cells, they may be an attractive model for developmentally young beta cells as they are, for example, present in patients with early-onset type 1 diabetes. The secretion of chemotactic signals may promote communication between SC-beta cells and immune cells, and non-insulin-producing cells possibly participate in the overall immune response and are thus capable of amplifying the immune response and further stimulating inflammation. We demonstrated that cytokine-treated SC-organoids secrete IL-32, which is considered a promising candidate for type 1 diabetes onset. This underlines the need to ensure the survival of SC-beta cells in an autoimmune environment such as that found in type 1 diabetes.

**Graphical abstract:**

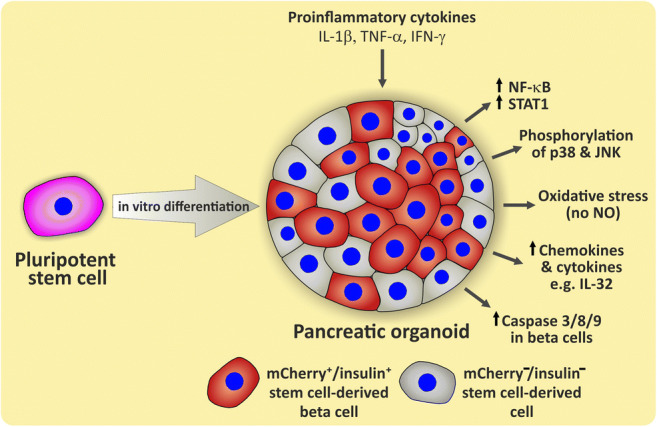

**Supplementary Information:**

The online version of this article 10.1007/s00125-022-05654-0 contains peer-reviewed but unedited supplementary material.

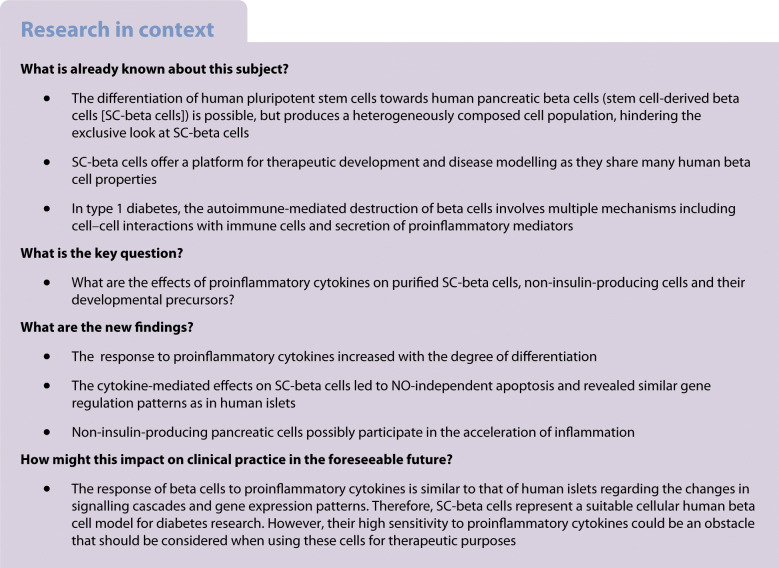



## Introduction

Type 1 diabetes is a chronic autoimmune disease characterised by loss of insulin production resulting from the selective and progressive destruction of beta cells. Activated immune cells infiltrate the islets of Langerhans and mediate their harmful effects through soluble cytotoxic mediators and cell–cell contacts. Proinflammatory cytokines, such as IL-1β, TNF-α and IFN-γ, are considered to be major mediators for the destruction of beta cells [1–3]. As a result of the cytokine stimulus various signal transducers, such as the transcription factors signal transducer and activator of transcription 1 (STAT1), NF-κB and interferon regulatory factor 1 (IRF-1) [3, 4], are activated, leading to oxidative stress, endoplasmic reticulum (ER) stress and apoptosis. ER stress, IL-1 and/or TNF can also induce the stress-signalling pathways c-Jun N-terminal kinase (JNK) and p38 mitogen-activated protein kinases (p38) [5–7]. In a vicious circle, cytokine exposure induces expression of MHC class I and II proteins and secretion of cytokines and chemokines such as chemokine (C-X-C motif) ligand 9/10 (CXCL9/10), CC-chemokine ligand 2/3/5/8 (CCL2/3/5/8) and others by beta cells, thereby promoting a destructive dialogue between immune cells and beta cells, resulting in acceleration of islet inflammation (insulitis) [8, 9].

The main cause of beta cell death is apoptosis. However, the mechanism of beta cell apoptosis and death in type 1 diabetes is not yet fully understood. This is partly caused by substantial species differences in the pathomechanism between rodents and humans [10, 11] and also because of the limited availability of human islets for research. Human pluripotent stem cells (hPSCs) can be differentiated into beta cells [12, 13]. Such stem cell-derived beta cells (SC-beta cells) are a promising donor-independent cellular model for research and drug development. Although SC-beta cells share many characteristics with human islet beta cells [13], they are phenotypically assigned to fetal perinatal beta cells due to their, in comparison with bona fide human beta cells, poorer glucose-stimulated insulin secretion, lack of biphasic insulin secretion and insufficient glucokinase and/or GLUT1/2 expression [14, 15]. SC-beta cells are also a possible source for cell replacement therapy of type 1 diabetes and type 2 diabetes [16, 17]. In type 1 diabetes in particular, the immune system represents a hurdle for cell replacement therapy because the immunity acquired against beta cell-specific antigens is retained after the body’s own beta cells are lost and this may cause another attack on transplanted SC-beta cells. In this environment with persistent autoimmunity an encapsulation of stem cell implants is thought to provide an immune protective environment while containing possible tumorigenic cells [18]. Soluble mediators, such as proinflammatory cytokines, could, however, overcome the encapsulation barrier [19]. So far only one pioneering study has dealt with the question of cytokine toxicity using heterogeneously composed stem cell-derived pancreatic organoids (SC-organoids) [20]. However, directed differentiation does not exclusively generate SC-beta cells but also other pancreatic and non-pancreatic cell populations [21]. In order to address the problem of heterogeneity, we have recently developed dual reporter hESC lines (SOX9 [SRY-box transcription factor 9]-GFP2/H-2K^K^ [H-2 class I histocompatibility antigen, K-K alpha chain], insulin [INS]-mCherry) [22]. This allowed us to assign the effects of our experiments to SC-beta cells in a highly specific manner and to differentiate them from other cell types.

## Methods

### Human cell culture

**Fig. 1:**
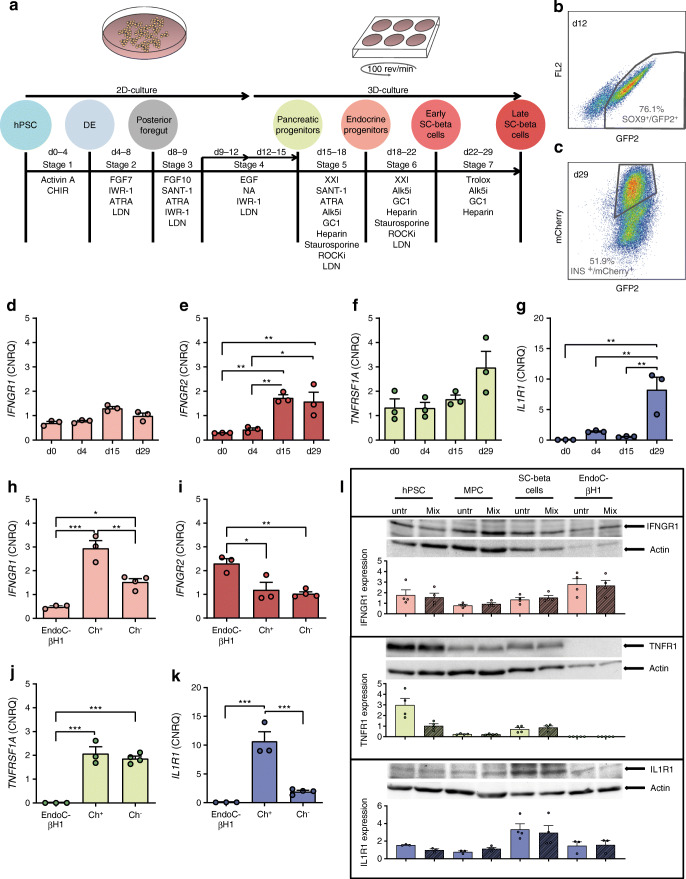
Cytokine receptor expression in human SC-beta cells and their progenitors. (**a**) Schematic presentation of the differentiation protocol used in this study. (**b**, **c**) Example flow cytometric dot plots of SOX9/GFP2-expressing cells on d12 of differentiation (**b**) and INS/mCherry-expressing cells on d29 of differentiation (**c**). (**d**–**g**) RT-qPCR analysis of cytokine receptor expressions of *IFNGR1* (**d**), *IFNGR2* (**e**), *TNFRSF1A* (**f**) and *IL1R1* (**g**) on d0 (hPSCs), d4 (DE), d15 (MPCs) and d29 (SC-organoids) of differentiation. Values are means ± SEM. *p<0.05, **p<0.01, ***p<0.001, *n* = 3. ANOVA plus Tukey’s post-test. (**h**–**k**) RT-qPCR analysis of *IFNGR1* (**h**), *IFNGR2* (**i**), *TNFRSF1A* (**j**) and *IL1R1* (**k**) receptor expression of sorted mCherry^+^ and mCherry^−^ cells on d28 of differentiation in comparison with EndoC-βH1 cells. Values are means ± SEM. *p<0.05, **p<0.01, ***p<0.001, *n* = 3–4. ANOVA plus Tukey’s post-test. (**l**) Representative western blots and densitometry analysis of IL1R1, IFNGR1 and TNFR1 expression in different developmental stages of untreated (untr) and 24 h cytokine-treated (Mix) cells. Protein expression is normalised to actin and values are means ± SEM. *n* = 3–4. Alk5i, Alk5 inhibitor; ATRA, all trans retinoic acid; Ch, mCherry; CHIR, CHIR99021; CNRQ, calibrated normalised relative quantity; FGF, fibroblast growth factor; FL2, fluorescence channel 2 (575 nm); GC1, thyroid hormone receptor beta-specific agonist GC1; IWR-1, Wnt pathway inhibitor IWR-1; LDN, LDN193189; NA, nicotinamide; ROCKi, ROCK inhibitor Y-27632; SANT1, Sonic hedgehog signalling inhibitor; XXI, gamma-secretase inhibitor XXi

Routine culturing of the mycoplasma-negative human reporter ESC lines HES-3 SC30 ICNC4 and HES-3 SC30 [22] and the parental cell line HES-3 (‘ESC’, ES Cell International [ESIBI], Singapore) was performed on Matrigel-coated (Corning, Amsterdam, the Netherlands) six-well plates using mTeSR1 medium (Stem Cell Technologies, Cologne, Germany). The culture of EndoC-βH1 cells was performed according to the standard protocol [23]. Differentiation was performed as described before [22] (Fig. 1a) and differentiation efficiency was monitored with the help of the reporter cell lines via flow cytometry (Fig. 1b, c). For further information, see the electronic supplementary material (ESM) Methods.

### Gene expression analysis, transcriptome analysis, western blot

For gene expression analysis, the cell line HES-3 SC30 ICNC4 was used. RNA was isolated using the Macherey- Nagel Nucleospin RNA Plus Kit (Macherey-Nagel, Düren, Germany). RevertAid H Minus M-MuLV Reverse Transcriptase (Thermo Fisher Scientific, Schwerte, Germany) and random hexamer primers (Thermo Fisher Scientific) were used to synthesise cDNA from 500–2000 ng of total RNA. SYBR Green-based quantitative PCR reactions were measured as described in detail in the ESM Methods. Primer sequences are listed in ESM Table 1.

Whole transcriptome microarray analysis was performed by the central facility ‘Genomics’ of the Hannover Medical School, as described in detail in the ESM Methods and ESM Fig. 1.

For western blot analysis, cells derived from HES-3 SC30 were used and protein expression was analysed as described in detail in the ESM Methods. Antibodies are listed in ESM Table 2.

### Flow cytometry and cell sorting

FACS was carried out by the central cell sorting facility of the Hannover Medical School. Flow cytometry and magnetic-activated cell sorting (MACS) were done as described in detail in the ESM Methods.

### Cytokine treatment, apoptosis assessment, and measurement of NO and ROS

Cells were treated with a common cytokine mix containing 185 U/ml TNF-α, 60 U/ml IL-1β and 14 U/ml IFN-γ (all ReliaTech, Wolfenbüttel, Germany) [1, 24] for 12 h, 24 h or 48 h in their respective differentiation or culture media. Apoptosis was assessed using the Caspase-Glo 3/7, the Caspase-Glo 8 or the Caspase-Glo 9 Assay Systems or the RealTime-Glo Annexin V Apoptosis Assay (all Promega, Walldorf, Germany) according to the manufacturer’s instructions. For details see the ESM Methods.

NO was measured as accumulated nitrite in the medium by the Griess reaction and intracellular reactive oxygen species (ROS) were estimated using the fluorescent probe dichlorofluorescein diacetate (DCFDA, Thermo Fisher Scientific), as described in detail in the ESM Methods. Definitive endoderm (DE) cells were derived from HES-3 and SC-organoids were derived from HES-3 SC30 ICNC4.

### ELISA IL-32

Secreted IL-32 from SC-organoids (derived from HES-3 SC30 ICNC4) or EndoC-βH1 cells was measured with the human IL-32 ELISA Kit (ELISAGenie, London, UK) according to the manufacturer’s instructions.

### Statistics

Data are expressed as means ± SEM unless stated otherwise. Statistical analyses were performed by GraphPad PRISM 8 (San Diego, CA, USA) using two-tailed paired Student’s *t* test or one-way ANOVA followed by Tukey’s post-test. For details, see the ESM Methods.

### Ethics approval

The author’s institution holds a license for the import and usage of human embryonic stem cells for the studies conducted in this manuscript (Robert Koch Institute, Berlin, Germany, AZ:1710-79-1-4-8E4).

## Results

To determine the responsiveness of the cells to the proinflammatory cytokines IL-1β, TNF-α and IFN-γ, we first analysed the expression of their corresponding receptors during the course of differentiation towards stem cell-derived organoids (SC-organoids) in comparison with the widely used human EndoC-βH1 beta cell line [25]. All receptors were expressed (Fig. 1d–g) and especially the expression of IL1 receptor 1 (IL1R1) increased significantly in SC-organoids (Fig. 1g). The insulin-producing SC-beta cells were purified with the help of an integrated mCherry reporter, whose expression is controlled by the insulin promoter. In a comparative analysis (Fig. 1h–k), especially *IL1R1* expression in mCherry^+^/insulin-producing SC-beta cells was increased compared with the mCherry^−^/non-insulin-producing population (Fig. 1k). The gene expression results could also be confirmed by western blot and densitometry analysis of the cytokine receptors IFN gamma receptor 1 (IFNGR1), IL1R1 and TNF receptor 1 (TNFR1) (Fig. 1l).

**Fig. 2:**
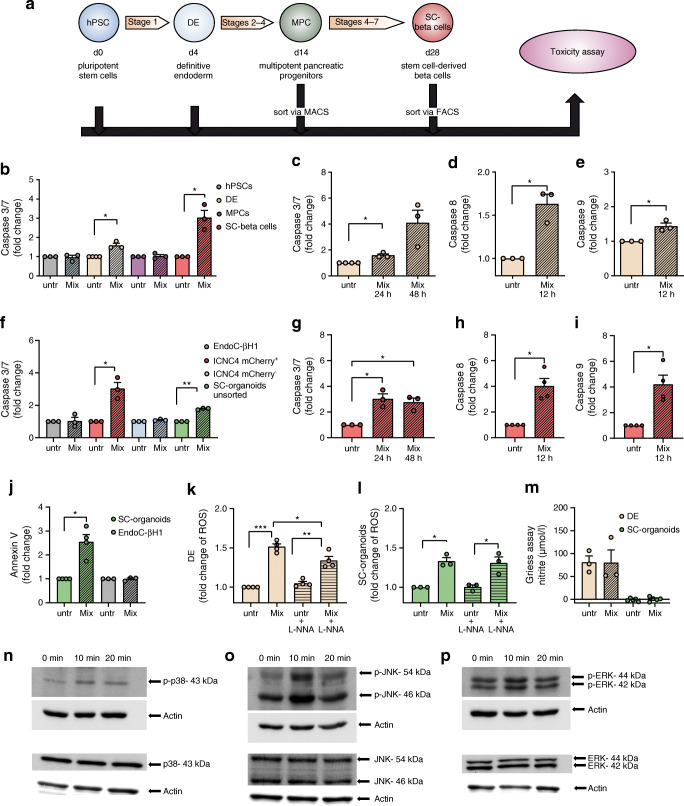
Assessment of cytokine toxicity and apoptosis signalling. (**a**) Schematic presentation of hPSC differentiation towards SC-beta cells and time points for toxicity tests. (**b**) Analysis of caspase 3/7 activation of 24 h cytokine-treated cells (Mix) compared with untreated cells (untr) on hPSCs (d0), DE (d4), MPCs (d14) and sorted SC-beta cells (d28). Values are means ± SEM. *p<0.05, *n* = 3. Two-tailed, paired Student’s *t* test. (**c**) Analysis of caspase 3/7 after 24 h and 48 h and caspase 8 (**d**) and 9 (**e**) activation (after 12 h) in cytokine-treated vs untreated DE cells. Values are means ± SEM. *p<0.05, *n* = 3. Two-tailed, paired Student’s *t* test. (**f**) Comparison of caspase 3/7 activation of cytokine-treated vs untreated EndoC-βH1-cells, sorted mCherry^+^ and mCherry^−^ cells of the cell line HES3 SC30 ICNC4 and unsorted pancreatic organoids of the cell line HES3 SC30. Values are means ± SEM. *p<0.05, **p<0.01, *n* = 3. Two-tailed, paired Student’s *t* test. (**g**) Analysis of caspase 3/7 after 24 h and 48 h and caspase 8 (**h**) and 9 (**i**) activation after 12 h in cytokine-treated vs untreated sorted SC-beta cells. Values are means ± SEM. *p<0.05, *n* = 3–4. Two-tailed, paired Student’s *t* test. (**j**) Confirmation of apoptosis assessment in SC-organoids and EndoC-βH1 cells by annexin V binding. Values are means ± SEM. *p<0.05 , *n* = 3–4. Two-tailed, paired Student’s *t* test. (**k**, **l**) Generation of ROS in cytokine-treated compared with untreated DE cells (**k**) and SC-organoids (**l**) with and without addition of L-NNA. Values are means ± SEM. *p<0.05, **p<0.01, ***p<0.001, *n* = 3–4. Two-tailed, paired Student’s *t* test. (**m**) Assessment of NO secretion via Griess assay in cytokine-treated vs untreated DE and SC-organoids. Values are means ± SEM, *n* = 3–4. (**n**–**p**) Western blot analysis of p38 (**n**), JNK (**o**) and ERK (**p**) phosphorylation

To assess the cytokine sensitivity of the cells, we investigated caspase 3/7 activation after a 24 h cytokine treatment in various developmental stages (Fig. 2a, b). In DE cells a 1.8-fold and in SC-beta cells a threefold increased caspase 3/7 activation could be observed upon incubation with proinflammatory cytokines (Fig. 2b). In DE cells, the caspase 3/7 activation observed after 24 h (Fig. 2b, c) increased further after a longer, 48 h cytokine exposure, albeit not to a significant extent (*p*=0.061) (Fig. 2c). Furthermore, a 1.6-fold caspase 8 (Fig. 2d) and a 1.4-fold caspase 9 (Fig. 2e) activation in DE cells could be determined after 12 h. We could not observe any activation of caspase 3/7 after 24 h in the reference human beta cell line EndoC-βH1. In stem cell-derived cells caspase 3/7 activation was almost exclusively limited to the mCherry^+^ population when compared with mCherry^−^ cells, which showed no significant increase (Fig. 2b). Unsorted SC-organoids, without a reporter gene in the insulin-locus (HES-3 SC30), showed a significant 1.8-fold increase in caspase 3/7 activity (Fig. 2f). The caspase 3/7 activation of mCherry^+^ cells observed after 24 h (Fig. 2b–g) remained at the same level after 48 h (Fig. 2g). Furthermore, we found strong caspase 8 and 9 activations (both fourfold) after 12 h of cytokine exposure (Fig. 2h, i). Additionally, apoptosis was confirmed by annexin V binding after 24 h of cytokine treatment in SC-organoids. Accordingly, no increase in annexin V binding was observed in EndoC-βH1 cells (Fig. 2j). Since DE cells and SC-beta cells were sensitive to cytokines, we estimated the overall generation of ROS in these cells after cytokine incubation (Fig. 2k–l). In order to determine the contribution of NO in the total amount of ROS detected by DCFDA oxidation, we added the nitric oxide synthase (NOS) inhibitor nitro-l-arginin (L-NNA). In DE cells the increase (1.5-fold) in total ROS generation after cytokine exposure was slightly reduced with the addition of L-NNA (Fig. 2k), unlike in SC-beta cells, whose increase in ROS generation (1.3-fold) was not influenced by L-NNA (Fig. 2l). In addition, we measured NO as accumulated nitrite in the medium by the Griess reaction and found no increase in NO in the supernatants of cytokine-treated compared with untreated cells after 24 h, either for DE cells or for unsorted SC-organoids (Fig. 2m). However, it is noticeable that the basic level of NO in DE cells was already somewhat elevated (Fig. 2m). Next, we investigated the induction of mitogen-activated protein-kinase (MAPK) signalling pathways by western blot analysis of JNK, p38 and extracellular-signal regulated kinase (ERK). Exposure to cytokines induced phosphorylation of JNK (Fig. 2o) and p38 (Fig. 2n) after 10 min, which was levelled off again already after 20 min. We did not observe any changes in ERK phosphorylation (Fig. 2p).

**Fig. 3:**
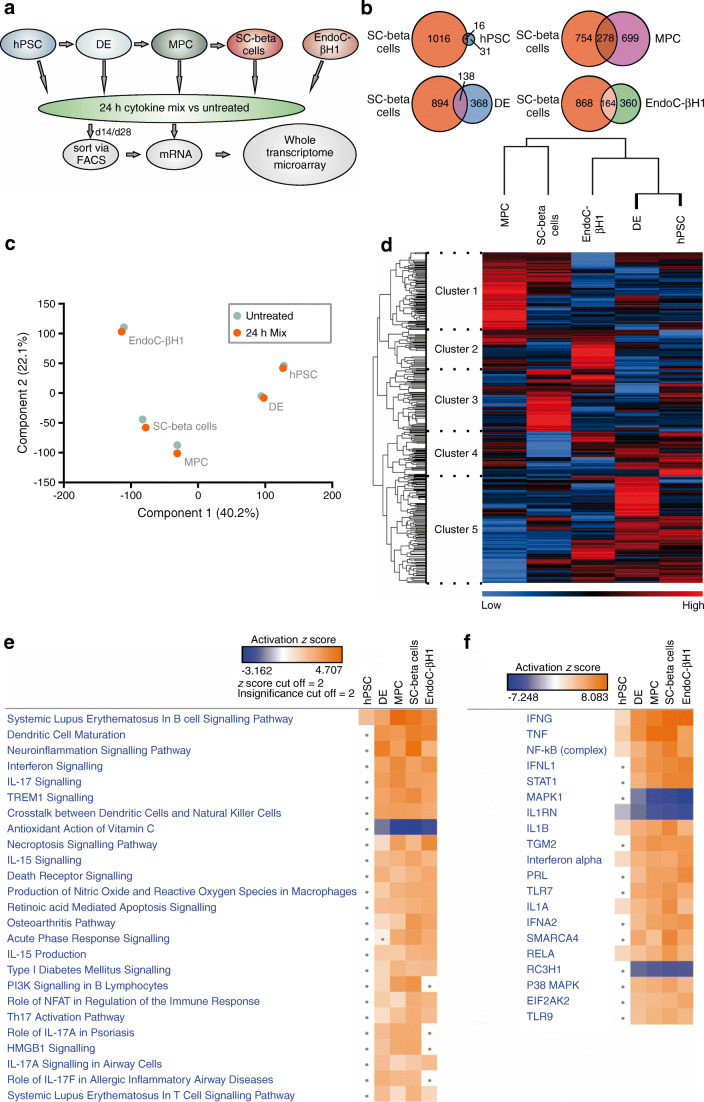
Analysis of transcriptome microarray data. (**a**) Schematic description of sample generation for the transcriptome analysis. (**b**) Number of genes jointly regulated (fold change ≥2) with SC-beta cells over the course of differentiation and in comparison with EndoC-βH1 cells, after a 24 h cytokine exposure. (**c**) PCA of the generated transcriptome data. (**d**) Hierarchical clustering of expression fold changes of cytokine-treated cells during the different stages of differentiation and EndoC-βH1 cells. (**e**) Comparative canonical pathway analysis of hPSCs (d0), DE (d4), MPCs (d14), SC-beta cells (d28) and EndoC-βH1 cells after cytokine treatment calculated by IPA. (**f**) IPA upstream analysis of cytokine-treated hPSCs, DE, MPCs, SC-beta cells and EndoC-βH1 cells. EIF2AK2, eukaryotic translation initiation factor 2 alpha kinase 2; HMGB1, high mobility group box 1; IFNA2, interferon alpha 2; IFNG, interferon gamma; IFNL1, interferon lamda 1; IL1A, interleukin 1 alpha; IL1B, interleukin 1 beta; NFAT, NFAT nuclear factor; PI3K,phosphatidylinositol 3-kinase; PRL, prolactin; RC3H1, ring finger and CCCH-type domains 1; RELA, RELA proto-oncogene, NF-kB subunit; SMARCA4, SWI/SNF related, matrix associated, actin dependent regulator of chromatin, subfamily a, member 4; TGM2, transglutaminase 2; Th17, T-helper 17; TREM1, triggering receptor expressed on myeloid cells 1

The changes at the gene expression level were determined using a whole transcriptome microarray. This was done for SC-beta cells and for different developmental stages and EndoC-βH1 cells (Fig. 3a). During the course of differentiation, the number of differentially regulated (fold change ≥2) genes increased and the number of intersections of genes differentially regulated in differentiating cells and SC-beta cells, respectively, also increased with their degree of differentiation (Fig. 3b). The intersection of differentially regulated genes of EndoC-βH1 cells and SC-beta cells was smaller compared with that of SC-beta cells and their progenitors. Likewise, the effect of cytokines further increased during differentiation as indicated by the fold changes of the 30 most up- and downregulated genes in the individual developmental stages (ESM Tables 3, 4). A principal component analysis (PCA) resulted in clustering of differentiation stages, demonstrating that the impact of differentiation on the overall transcriptome was higher compared with the impact of cytokine treatment (Fig. 3c). The gene regulation profiles and their overlaps became visible in the heatmap analysis (Fig. 3d). The classification of the different gene clusters can be found in ESM Table 5. Generally, Qiagen Ingenuity Pathway Analysis (IPA) revealed activation of canonical signalling pathways involved in immune response, inflammation and autoimmune diseases (Fig. 3e). IPA upstream analysis (Fig. 3f) served as a positive control by pointing to the cytokines we used in our analysis and suggested pathways affected in treated cells. Strikingly, regulation of the NF-κB, STAT1 and p38 MAPK signalling pathways was predicted.

**Fig. 4:**
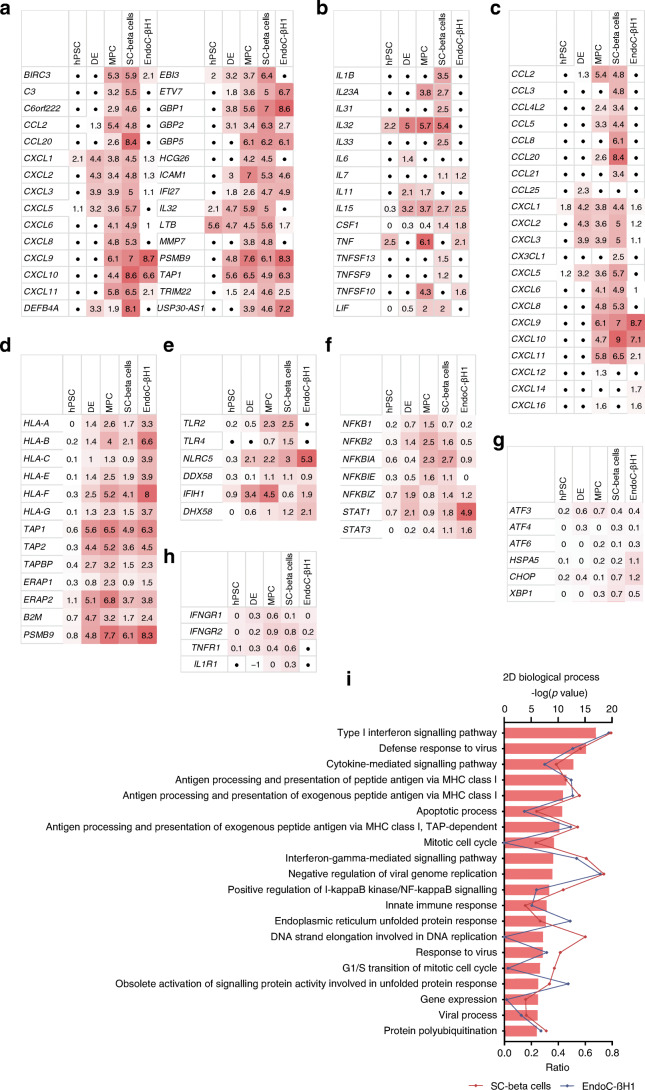
Summary of differentially regulated genes and pathways. (**a**) Top 30 jointly with SC-beta cells upregulated (fold change ≥2) genes of hPSCs, DE, MPCs and EndoC-βH1 cells (represented as log_2_ fold change; black circle, below threshold) after a 24 h cytokine treatment. Comparative tables of upregulated cytokines (**b**), chemokines (**c**), members of the MHC class I family (**d**), NF-κB- and STAT1-pathway-related genes (**e**), PRRs (**f**), typical ER stress markers (**g**) and cytokine receptors (**h**) after 24 h of cytokine treatment (represented as log_2_ fold change; black circle, below threshold). (**i**) 2D enrichment analysis of genes regulated in EndoC-βH1 and SC-beta cells according to the GO term ‘biological process’. The ratio represents the number of regulated genes related to the different biological processes

In the top 30 jointly regulated genes (Fig. 4a), we searched for genes that were upregulated in SC-beta cells, as well as in at least one other condition, in order to find further genes of interest and a possible conserved pattern. We noticed a number of chemokines, as well as genes of the interferon-induced guanylate-binding protein (GBP) family, genes related to class I antibody processing (*TAP1*, *PSMB9*), an inducer of the inflammatory response system (*LTB*) and the relatively unknown cytokine *IL32*. In total, 11 human cytokines were upregulated in our record (Fig. 4b) and 17 human chemokines (Fig. 4c). In addition, we found a number of upregulated genes that are related to the MHC class I family (Fig. 4d), the activation of the key transcription factors NF-κB and STAT1 (Fig. 4e) and pattern recognition receptors (PRRs) (Fig. 4f). We did not observe any regulation of cytokine receptors (Fig. 4h) or typical ER stress markers (*ATF3*, *ATF4*, *ATF6*, *CHOP* [also known as *DDIT3*], *HSPA5*, *XBP1*) in the microarray data. A weak regulation of *CHOP* and *HSPA5* could only be detected in EndoC-βH1 cells (Fig. 4g, ESM Fig. 2). A 2D enrichment analysis indicated significantly higher fold changes of genes related to the biological processes ‘cytokine-mediated signalling cascades’, ‘antigen processing’ and ‘immune response and ER stress’ (according to the gene ontology [GO] database) in EndoC-βH1 and SC-beta cells (Fig. 4i, ESM Table 6). The number of regulated genes (ratio) in the corresponding GO terms was partially similar for SC-beta cells and EndoC-βH1 cells, but there were also differences, e.g. with regard to mitosis and cell cycle (Fig. 4i). Overall, a comparison of our transcriptome analysis with RNA sequencing (RNA-seq) data from cytokine-treated (48 h) human islets [26] showed similarities between SC-beta cells and human islets (ESM Tables 7–11, ESM Fig. 3).

**Fig. 5:**
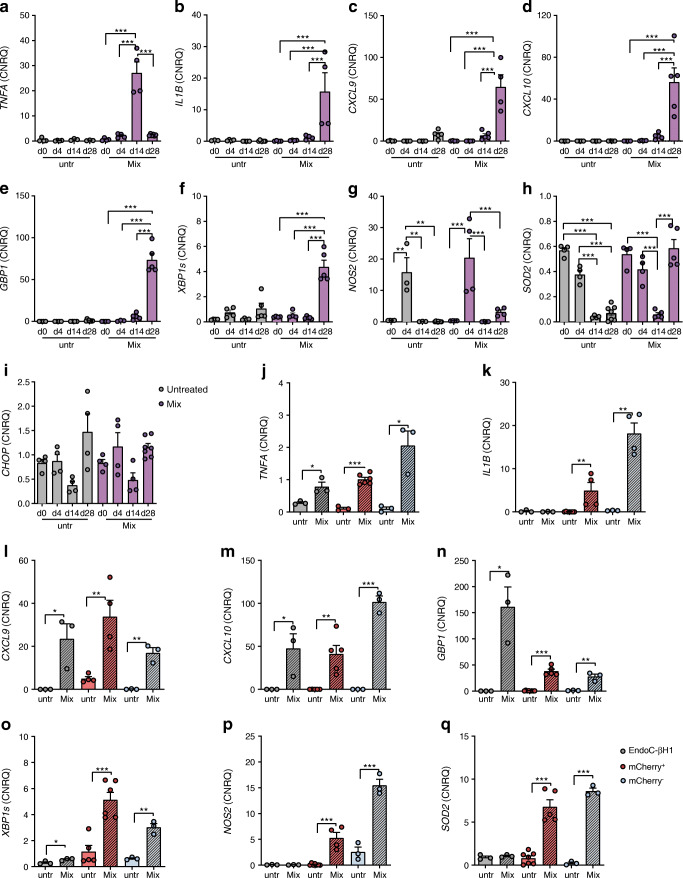
Validation of candidate gene expression. (**a**–**i**) RT-qPCR gene expression analysis of *TNFA* (**a**), *IL1B* (**b**), *CXCL9* (**c**), *CXCL10* (**d**), *GBP1* (**e**), *XBP1s* (**f**), *NOS2* (**g**), *SOD2* (**h**) and *CHOP* (**i**) in 24 h cytokine-treated (Mix) compared with untreated (untr) cells during the differentiation process. Values are means ± SEM. **p<0.01, ***p<0.001, *n* = 3–7. ANOVA plus Tukey’s post-test. (**j**–**q**) Comparative RT-qPCR gene expression analysis of *TNFA* (**j**), *IL1B* (**k**), *CXCL9* (**l**), *CXCL10* (**m**), *GBP1* (**n**), *XBP1s* (**o**), *NOS2* (**p**) and *SOD2* (**q**) in cytokine-treated vs untreated mCherry^+^/insulin-producing and mCherry^−^/non-insulin-producing stem cell-derived pancreatic cells and EndoC-βH1 cells. Values are means ± SEM. *p<0.05, **p<0.01, ***p<0.001, *n* = 3–9. ANOVA plus Tukey’s post-test. Data are represented as calibrated normalised relative quantity (CNRQ) after normalisation with qBase plus

Alongside, we reviewed gene expression changes of candidate genes using real-time quantitative PCR with reverse transcription (RT-qPCR) in order to verify the technical stability of the microarray. These genes comprised the cytokines *IL32*, *TNFA* (at day 28 [d28] just below the detection threshold of the microarray) and *IL1B*; the chemokines *CXCL9* and *10*, and *GBP1*; the ER stress markers *XBP1s* and *CHOP*; and *NOS2* and *SOD2* as indicators of cellular response to oxidative stress. First, we measured the gene expression changes during the course of differentiation, comparing untreated cells and cytokine-treated cells (Fig. 5a–i). *TNFA* was most strongly expressed by multipotent pancreatic progenitors (multipotent pancreatic progenitor cells [MPCs], d14) after cytokine incubation, whereas *IL1B*, *CXCL9*, *CXCL10* and *XBP1s* were most pronounced in the cytokine-treated SC-beta cells. *NOS2* and *SOD2* were only upregulated in SC-beta cells after cytokine treatment, but we found a very high initial level of expression for *NOS2* and *SOD2* in DE cells, although there was no upregulation of either. Besides the regulation of *XBP1s*, we could not detect any regulation of other typical ER stress markers in SC-beta cells (ESM Fig. 2). Next, we compared the basal expression and upregulation of the genes in mCherry^+^/SC-beta cells with mCherry^−^/non-insulin-producing cells and EndoC-βH1 cells (Fig. 5j–q), which revealed the regulation of all considered candidate genes also in non-insulin-producing cells.

**Fig. 6:**
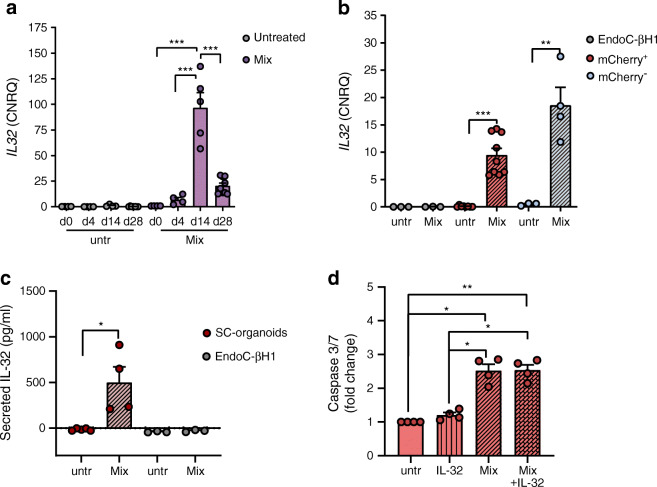
IL-32 expression and secretion in SC-organoids and IL-32 involvement in apoptosis. (**a**) RT-qPCR expression analysis of *IL32* in 24 h cytokine-treated (Mix) compared with untreated (untr) cells during the differentiation process. Values are means ± SEM. ***p<0.001, *n* = 3–7. ANOVA plus Tukey’s post-test. (**b**) Comparative RT-qPCR expression analysis of *IL32* in 24 h cytokine-treated (Mix) vs untreated (untr) mCherry^+^/insulin-producing and mCherry^−^/non-insulin-producing stem cell-derived pancreatic cells and EndoC-βH1 cells. Values are means ± SEM. **p<0.01, ***p<0.001, *n* = 3–9. ANOVA plus Tukey’s post-test. Data are represented as calibrated normalised relative quantity (CNRQ) after normalisation with qBase plus. (**c**) IL-32 secretion detected by ELISA in supernatants from unsorted SC-organoids and EndoC-βH1 cells that were untreated (untr) or cytokine-treated for 24 h (Mix) in each case. Values are means ± SEM. *p<0.05, *n* = 3–5. Two-tailed, unpaired Student’s *t* test. (**d**) Activation of Caspase 3/7 in SC-organoids after a 24 h exposure to IL-32 alone or in addition to the cytokine mix (Mix) compared with untreated (untr) cells. Values are means ± SEM. *p<0.05, **p<0.01, *n* = 4. Repeated measures (RM) ANOVA with Geisser–Greenhouse correction plus Tukey’s post-test

The cytokine *IL32*, which we noticed in the group of the top 30 jointly regulated genes with SC-beta cells in all differentiation stages (Fig. 4a), was most strongly expressed in MPCs (d14) treated with cytokines for 24 h (Fig. 6a). We found almost no expression of this cytokine in untreated cells of all types. In a comparative analysis, cytokines induced the expression of *IL32* most in the non-insulin-producing population. In our conditions, EndoC-βH1 cells did not express *IL32* after a 24 h cytokine treatment (Fig. 6b). When examining the medium supernatants from SC-organoids in comparison with EndoC-βH1 cells, we found IL-32 secretion only in supernatants of 24 h cytokine-treated SC-organoids (Fig. 6c). With regard to apoptosis, no significant increase in caspase 3/7 activation could be detected by exposure to IL-32 alone, or in combination with the cytokine mix (Fig. 6d).

## Discussion

The pathogenesis of autoimmune diabetes involves activation of a variety of immune cells and secretion of inflammatory mediators, which leads to the decrease of beta cell mass in patients with type 1 diabetes. In this study, we exposed hPSCs, DE cells, purified MPCs and SC-beta cells to proinflammatory cytokines and determined transcriptional changes and the mode of cell death. We observed the respective cytokine receptor expressions in all of these cell types. The strong expression of *IL1R1* in purified SC-beta cells is of particular interest, since human islet beta cells are known to be particularly sensitive to IL-1β-mediated beta cell destruction [27–30].

According to our analysis, the cellular response to proinflammatory cytokines increased with the degree of differentiation of the cells. Using our reporter cell line, we were able to differentiate between insulin-producing SC-beta cells and non-insulin-producing cells. A 24 h treatment with proinflammatory cytokines led to apoptosis of SC-beta cells, but not of the non-insulin-producing population. Similarly, SC-beta cells were more sensitive to cytokines than the human beta cell line EndoC-βH1, which is known for its delayed cytokine sensitivity mode [10, 31]. However, it should be noted that the sensitivity of EndoC-βH1 cells to proinflammatory cytokines depends on the composition of the cytokine mix used as well as the cultivation conditions. A higher cell density can lead to a higher sensitivity [32]. Additionally, EndoC-βH1 pseudoislets showed earlier and greater sensitivity to cytokine toxicity than EndoC-βH1 monolayers [31]. Importantly, human islets require cytokine exposure for several days to induce functional impairment and apoptosis [33]. We therefore assume that SC-beta cells with their immature phenotype are even more sensitive to cytokines than adult beta cells.

Apoptosis in SC-beta cells was mediated both by activation of the caspase 8-mediated extrinsic apoptosis signalling pathway likely triggered by TNF-α receptor binding, and by activation of the caspase 9-mediated intrinsic pathway. The observed low *NOS2* induction in SC-beta cells does not appear to be sufficient to produce NO to any significant extent after 24 h, and therefore it excludes the role of NO in the activation of early cytokine toxicity in these cells. This is in line with observations made in cytokine-treated EndoC-βH1 cells [10, 31, 34] and a minor role of NO in cytokine toxicity in human islets [27, 35, 36]. The induction of SC-beta cell apoptosis is more likely to take place via FAS-associated death domain (FADD) and/or stress-signalling pathways, such as JNK or p38, whose activation was detected shortly after cytokine addition. Moreover, induction of ROS generation after 24 h of cytokine exposure in SC-beta cells might also activate the intrinsic apoptosis signalling pathway [37] and participate in the rapid induction of apoptosis, additionally to ROS-mediated damage of cellular components. Thus we detected many similarities in the common mechanisms of cytokine toxicity between SC-beta cells, EndoC-βH1 cells [10, 31, 38] and human islets [26]; however, differences in time-line and magnitude of response are also apparent.

Analysis of the microarray data revealed the central importance of the NF-κB and STAT1 signalling pathways for the cellular effects, resulting in the expression of PRRs, MHC-I genes and a large range of cytokines and chemokines. In human islets, EndoC-βH1 cells and pancreases of recently diagnosed type 1 diabetes patients, the activation of NF-κB was shown to be significantly lower than in rodent beta cells and not primarily responsible for cytokine-mediated beta cell death [31, 39]. Using gene set enrichment analysis (GSEA) and analysis of enriched pathways, we were able to uncover a clear relation to cytokine-induced, inflammatory and immunomodulatory signalling pathways. The differential regulation of many genes in SC-beta cells often developed in progenitor stages and generally increased with the degree of differentiation. In conclusion, the progenitor populations are also potentially capable of communication with the immune system, although cytokine toxicity is less pronounced in these cells. Induction of cytokines and chemokines in cytokine-treated SC-beta cells was particularly striking. We detected a total of 12 cytokines, including *IL32*, as well as 17 differentially regulated chemokines, including *CXCL9* and *CXCL10*, the secretion of which has been already demonstrated in cytokine-treated pancreatic organoids derived from human induced pluripotent stem cells (iPSCs) and/or human islets [20, 26]. We observed a stage-dependent increase of *IL1B* and *TNFA* expression. *IL1B* and *TNFA* expression of SC-beta cells is consistent with findings for human islets [26]. Changes in gene expression do not necessarily result in secretion of bioactive proteins though. The secretion of IL-32, however, was verified in our study. Although we could not observe any *IL32* expression in EndoC-βH1 cells after a 24 h cytokine exposure under present conditions, a cytokine-induced increase in *IL32* gene expression has already been demonstrated for these cells in other studies [40]. IL-32 has only recently been identified as an early biomarker in the blood of children from type 1 diabetes-risk families even before autoantibody appearance and diabetes manifestation [40]. IL-32 is known to promote the expression of chemokines and proinflammatory cytokines such as TNF-α and IL-6 in human and murine immune cells [41]. An increased expression of IL-32 as a result of signals via Toll-like receptor 2 (TLR2), TLR3 and TLR4 has also been shown in human fibroblast-like synoviocytes [41, 42]. In addition, the involvement of IL-32 in the development of other autoimmune diseases, such as rheumatoid arthritis, has already been described [42]. The role of IL-32 in type 1 diabetes is still unclear, but it is possible that IL-32 might be crucially involved in the pathogenesis of autoimmune diabetes [43]. We did not detect a significant IL-32-induced increase in apoptosis in SC-organoids. In EndoC-βH1 cells IL-32 did not appear to directly affect survival of the cells [40]. Thus, IL-32 seems to participate in activating immune cells rather than directly damaging beta cells [40]. In any case, further studies on the role of IL-32 in the development of type 1 diabetes would be worthwhile.

Although our observations are consistent with previous analyses of gene regulation in human islets, it is noteworthy that only one point in time (24 h cytokine exposure) was examined in our study. It is possible that the dynamic regulation of gene expression of relevant cytokine-modulated genes was not recorded by our analysis at other times. For example, the increase in expression of *XBP1s* indicates ER stress, but we were not able to determine any regulation of *CHOP* or other typical ER stress markers after 24 h. According to GO terms, ER stress and unfolded protein response (UPR)-associated genes were regulated though. In another study on iPSC-derived organoids, ER stress occurred only after 48 h of cytokine treatment [20]. Therefore, we suggest that we were only able to depict the beginning of ER stress, which could, however, be increased over time. In conclusion, ER stress does not seem to be of primary importance for the rapid induction of apoptosis, but may play a greater role at later time points. It should also be mentioned here that only three pooled biological replicates were used to determine the microarray data. Increasing the number of biological replicates could therefore lead to clearer results. The stability of our microarray data, however, could be confirmed by the RT-qPCR validation of the candidate genes.

Overall, SC-beta cells are characterised by a similar pattern of differential gene regulation after cytokine exposure as human islets [26]. SC-beta cells are sensitive to cytokines; however, their time-response and sensitivity mode seem to be stronger than that of mature beta cells. The underlying mechanisms of apoptosis induction show many similarities, but require further investigation. We therefore believe that SC-beta cells can be a new attractive in vitro model for human beta cell studies. The immature phenotype of SC-beta cells could represent an interesting model for early-onset type 1 diabetes (EO-T1D). EO-T1D, marked by a fulminant manifestation of type 1 diabetes, is assigned in small children significantly younger than 3 years of age, in whom type 1 diabetes has already been diagnosed [44, 45]. In contrast, patients with type 1 diabetes manifestation in adulthood can be assigned a slow progressive course [46]. Beta cell loss and disease progression in autoimmune diabetes seem therefore to be age-related: the younger the patient is when the diagnosis is made, the faster and more intensive the disease progression is, and vice versa [47, 48].

Another interesting aspect of our study is a possible participation of non-insulin-producing pancreatic cells in the acceleration of inflammation. So far, little attention has been paid to non-insulin-producing pancreatic cells and their role in the immune response. A recently published RNA-seq analysis on mouse islets revealed the responsiveness of all endocrine cell types to cytokines despite the selective destruction of beta cells only [49]. In view of the heterogeneity of the cell population obtained after differentiation, it is an invaluable advantage to be able to differentiate between insulin-producing and non-insulin-producing cells when assessing the question of cell type-specific effects. The observed cytokine and chemokine expression of non-insulin-producing cells suggests a possible participation of these cells in the attraction of immune cells and in the destruction process of beta cells. Our data indicate that the cytokine signalling pathways are apparently also activated in these cells, though they are less prone to cytokine toxicity than SC-beta cells. We can therefore conclude that, with the approach of cell replacement therapy using SC-organoids, there may be the possibility of increased attraction of immune cells by SC-beta cells and non-insulin-producing cells. Furthermore, cytokine-mediated SC-beta cell death could represent an obstacle, especially in an environment with persistent autoimmunity.

## Supplementary information

ESM 1 (PDF 1.70 mb)

ESM 2 (XLSX 1.81 mb)

## Data Availability

The datasets generated during and/or analysed during the current study are available from the corresponding author on reasonable request.

## Abbreviations

CHOP: C/EBP homologous protein
d: Day
DCFDA: Dichlorofluorescein diacetate
DE: Definitive endoderm
EO-T1D: Early-onset type 1 diabetes
ER: Endoplasmic reticulum
ERK: Extracellular-signal regulated kinase
GO: Gene ontology
hPSC: Human pluripotent stem cell
IFNGR1: IFN gamma receptor 1
IL1R1: IL1 receptor 1
INS: Insulin
IPA: Qiagen Ingenuity Pathway Analysis
iPSC: Induced pluripotent stem cell
JNK: c-Jun N-terminal kinase
L-NNA: Nitro-l-arginine
MACS: Magnetic-activated cell sorting
MAPK: Mitogen-activated protein-kinase
MPC: Multipotent pancreatic progenitor cell
p38: p38 mitogen-activated protein kinases
PCA: Principal component analysis
PRR: Pattern recognition receptor
RNA-seq: RNA sequencing
ROS: Reactive oxygen species
RT-qPCR: Quantitative PCR with reverse transcription
SC-beta cell: Stem cell-derived beta cell
SC-organoids: Stem cell-derived pancreatic organoids
SOX9: SRY-box transcription factor 9
STAT1: Signal transducer and activator of transcription 1
TLR: Toll-like receptor
TNFR1: TNF receptor 1
