# Supplementary material for: Proinflammatory cytokines induce rapid, NO-independent apoptosis, expression of chemotactic mediators and interleukin-32 secretion in human pluripotent stem cell-derived beta cells
Source: Diabetologia. 2022 Feb 5;65(5):829–43. doi: 10.1007/s00125-022-05654-0 (PMC8960637; doi:10.1007/s00125-022-05654-0)
Supplement: Supplementary file 1 — (PDF 1.70 mb) [file 125_2022_5654_MOESM1_ESM.pdf]

ELECTRONIC SUPPLEMENTARY MATERIAL (ESM)

Proinflammatory cytokines induce rapid, NO-independent apoptosis, expression of chemotactic mediators and interleukin-32 secretion in human pluripotent stem cell-derived beta cells

Rabea Dettmer[1], Isabell Niwolik[1], Karsten Cirksena[1], Toshiaki Yoshimoto[1,2], Yadi Tang[1], Ilir Mehmeti[1], Ewa Gurgul-Convey[1] and Ortwin Naujok[1]

[1] Institute of Clinical Biochemistry, Hannover Medical School, Hannover, Germany

[2]Department of Digestive and Transplant Surgery, Tokushima University, Tokushima, Japan

Corresponding author:

Ortwin Naujok

ESM Methods

Human cell culture

SC-beta cells and pancreatic organoids, respectively, were differentiated from hPSC and transgenic clonal cell lines (HES-3, HES3 SC-30, HES-3 SC30 ICNC4) using a 3D production protocol, which is optimized for the production of SOX9-MPCs and produces an average MPC content of over 70% and a stable average SC-beta cell content of over 40% [1] (Fig 1b, c). HES-3 is the parental hESC line, HES-3 SC30 harbors a knock-in of GFP2/H2K^k in the *SOX9*-Locus (MPC-stage). HES-3 SC30 ICNC4 additionally contains a mCherry knock-in into the *INS*-Locus (endocrine stage). These reporter cell lines allowed the purification of MPCs and SC-beta cells via FACS and MACS and enabled monitoring of

differentiation via flow cytometry (Fig 1b, c). The reporter gene insertions were mediated by the CRISPR/Cas9 and homologous recombination technology [1].

Gene expression analysis

For gene expression analysis, technical triplicate reactions were carried out in a 2-step PCR in a ViiA7 real-time PCR cycler (Thermo Fisher Scientific, Schwerte, Germany). SYBR Green-based qPCR reaction were measured with cDNA diluted to 2.5-5 ng/µl and the GoTaq® qPCR Master Mix (Promega, Walldorf, Germany). Data were normalised against the geometric mean of the housekeeping genes *G6PD*, *TBP* and *TUBA1A* using qBase plus V. 2.3 (Biogazelle, Zwijnaarde, Belgium) and presented as relative gene expression termed CNRQ (Calibrated Normalised Relative Quantity). The RT-qPCR validation of the candidate genes for SC-beta cells was carried out using biological replicates other than those used for the microarray data.

Transcriptome Analysis

The Whole Human Genome Oligo Microarray Kit 4x44K v2 (G4845A, design ID 026652, Agilent Technologies, Santa Clara, United States) was used to analyze gene expression. 150 ng RNA of three pooled biological replicates for untreated cells and 24 h cytokine-treated cells were used as input. RNA was prepared from hPSC (d0, derived from HES-3), definitive endoderm (DE; d4, derived from HES-3), GFP2/H2-K^{K+}-sorted multipotent pancreatic progenitor cells (MPC) (d14, derived from HES-3 SC30), mCherry^{+}-sorted SC-beta cells (d28, derived from HES-3 SC30 ICNC4) [1] and EndoC-βH1 cells. The synthesis of the Cy3-labeled cDNA was carried out in 75% reaction volume of the Low Input Quick Amp Labeling Kit One-Color (# 5190-2305, Agilent Technologies) according to the manufacturer's instructions. CDNA fragmentation, hybridization and washing steps were carried out in accordance with the procedure described in One-Color Microarray-Based Gene Expression Analysis Low Input Quick Amp Labeling Protocol V6.7. The analysis was carried out with

the Agilent Micro Array Scanner G2565CA. Data was extracted with Feature Extraction Software V10.7.3.1 and analysis of the received data was done with Perseus Software V. 1.6.15.0 (https://maxquant.net/perseus/) and Ingenuity Pathway Analysis (IPA, Qiagen, Hilden). The procedure of data processing is shown in the ESM Fig 3.

Western Blot

Cells were sonicated in PBS containing protease inhibitor mixture (Roche Diagnostic, Mannheim, Germany). Protein amount was measured using a BCA assay (Thermo Fisher Scientific, Schwerte, Germany). Separation was carried out by SDS-PAGE using 40 µg of total protein, followed by blotting onto polyvinylidene fluoride (PVDF) membranes. Subsequently the membranes were blocked using 5% nonfat dry milk in PBS plus 0.1% Tween 20. Membranes were incubated with primary antibodies (Supplementary table 1) overnight at 4°C and washed. Then the membranes were incubated with a peroxidase-labeled secondary antibody for 1 h. Actin was used as a loading control. Protein bands were visualized using an enhanced-chemiluminescence detection kit (GE Healthcare Europe, Solingen, Germany) and captured by the INTAS chemiluminescence-detection system (Intas Science Imaging Instruments, Göttingen, Germany). For densitometric analysis, protein expression was normalised to actin.

Cytokine treatment and apoptosis assessment

hPSCs (d0, HES-3), definitive endoderm cells (DE) (d4, derived from HES-3), multipotent pancreatic progenitors (MPC) (d14, derived from HES-3 SC30), SC-beta cells (d28, derived from HES-3 SC30 ICNC4), SC-organoids (d28, derived from HES-3 SC30) and the control cell line EndoC-βH1, were treated with a common cytokine-mix [2, 3] containing 185 U/ml TNF-α, 60 U/ml IL-1β and 14 U/ml IFN-γ (all Reliatech, Wolfenbüttel, Germany) for 12 h, 24 h or 48 h in their respective differentiation- or culture media. EndoC-βH1 cells were seeded as monolayers in a density of 20000 cells/cm^2. Apoptosis was determined using the

Caspase-Glo 3/7 Assay System, the Caspase-Glo 8 Assay System, the Caspase-Glo 9 Assay System and the RealTime-Glo Annexin V Apoptosis Assay (all from Promega, Walldorf, Germany) according to the manufacturer´s instructions in a luminometer (Glomax Plus, Promega, Walldorf, Germany). Caspase activation in MPC and SC-beta cells were tested after cell sorting. To examine the influence of IL-32 on cytokine toxicity of SC-beta cells, IL-32 (Abcam, Berlin, Germany) was used either alone or supplemented to the cytokine mix at a concentration of 100 ng/ml (≤1.000 EU/µg). This was performed on unsorted SC organoids derived from HES-3 SC30 ICNC4. The CellTiter-Fluor™ Assay (Promega, Walldorf, Germany) was used for data normalization.

Measurement of NO and ROS

For measurement of NO by the Griess reaction, briefly, 50 µL of supernatant was applied to a 96-well microtiter plate. Each sample was assayed in triplicate. 50 µl of a 1:1 mixture of sulfanilic acid (1%) in 0.1 M HCl and N-ethylenediamine hydrochloride (0.1%) was added to the supernatant. After mixing, nitrite production was determined spectrophotometrically at an absorbance of 562 nm in a microtiter plate. Freshly prepared sodium nitrite standards were used to normalize the assay reactivity and associated absorbance.

For estimation of intracellular ROS, cells were incubated with DCFH-DA for 30 min at 37°C and 5% CO_2 in culture medium. The nitric oxide synthase (NOS) inhibitor L-NNA (nitro-L-arginin, Sigma-Aldrich, Taufkirchen, Germany) was added (1 µM) to exclude NO in the total amount of ROS. Then cells were incubated with cytokines for 24 h, dissociated into single cells and analyzed. SC-beta cells were analyzed by flow cytometry in a CyFlow ML flow cytometer (SysmexPartec, Münster, Germany), DE cells were analyzed in a microplate reader (Synergy™ Mx, BioTek, Bad Friedrichshall, Germany).

Flow cytometry

For flow cytometric analysis the cells were washed with PBS and dissociated using trypsin/EDTA (T/E). Pancreatic organoids from 3D culture were pre-incubated with gentle cell dissociation reagent (Stem Cell Technologies) for 15 min at 37°C and finally dissociated using T/E for additional 10 min. Antibody stainings were performed following standard protocols. Samples were analyzed on a CyFlow ML flow cytometer (SysmexPartec, Münster, Germany).

Cell sorting

For Magnetic associated cell sorting (MACS) dissociated single cells in PBE buffer (PBS, pH 7.2, 0.5 % BSA, and 2 mM EDTA) were incubated for 15 min with anti H2-K^k magnetic microbeads (Miltenyi-Biotec, Bergisch Gladbach, Germany) on ice and were sorted by an AutoMACS Pro (Miltenyi-Biotec, Bergisch Gladbach, Germany).

Statistics and experimental procedures

Since we could not make any reliable predictions about the effect size of our experiments, we did not calculate any kind of power analysis. Instead we performed the minimum number of 3 independent biological replicates per experiment. If a trend resulted from these experiments, repetitions of the experiment were performed to either confirm or refuse the null hypothesis. The number of tests can be found in the legend for each figure, as well as the statistical test used.

Information about the number of independent biological replicates (n) is provided in the respective figure legends or in the figures itself. No technical replicates are included. Outliers were excluded if technical reasons were responsible for a significantly different measured value and if individual values were significantly different from the median or mean of a biological group.

For single comparisons, the paired, two-tailed *Student's* t-test was performed. For multiple comparisons ANOVA plus *Tukey's* post-test was used. For statistical hypothesis testing, specifically in null hypothesis significance testing, we used the *p* value method. Significance levels were defined as $p < 0.05$, (5% α-value); $p < 0.01$ (2% α-value); $p < 0.001$ (0.1% α-value). If the *p* value was less than the chosen significance level (α = less than 5%), we rejected the null hypothesis. Only in this case was the term used 'significantly' or 'significant' when describing the experimental results. Statistical tests were not carried out if the questions were irrelevant for the key statements of this study.

Samples from human embryonic stem cells were allocated to two different groups. Next all treatment regimens were individually compared against untreated control groups or EndoC-βH1 cells. No masking was used during group allocation, data collection and/or data analysis.

ESM Table 1: Primer pairs for gene expression analysis.

Gene	Primer Sequence 5'-3'	Accession #
TNFA	Fw: AGA ACT CAC TGG GGC CTA CA<br>Rev: AGG AAG GCC TAA GGT CCA CT	NM_000594
IL1B	Fw: GCT CGC CAG TGA AAT GAT GG<br>Rev: GGT GGT CGG AGA TTC GTA GC	NM_000576.3
IL32	Fw: CAG GAA GAC TGC GTG CAG AA<br>Rev: TCT ATG GCC TGG TGC ATT CG	NM_001012631.4
GBP1	Fw: AGG AGA AAA AGA ACA GAC AAG GGA<br>Rev: AGA GCT TCT GGA TTC GCC ATC	NM_002053
XBP1s (spliced)	Fw: TGC TGA GTC CGC AGC AGG TG<br>Rev: GCT GGC AGG CTC TGG GGA AG	NM_001079539.2
CXCL9	Fw: GCC ATC CTG CCC ATA ACA<br>Rev: GAG GGC AAG AGC CAC AGT AT	NM_002416
CXCL10	Fw: GTG GCA TTC AAG GAG TAG CTC<br>Rev: GCC TTC GAT TCT TGG ATT CAG	NM_001565
NOS2	Fw: AGC GGG ATG ACT TTC CAA<br>Rev: AGG CAA GAT TTG GAC CTG CA	NM_000625
SOD2	Fw: GCT CCG GCT TTG GGG TAT CT<br>Rev: TTG TTC ACG TAG GCC GCG T	NM_000636.4
G6PD	Fw: AGG CCG TCA CCA AGA ACA TTC A<br>Rev: CGA TGA TGC GGT TCC AGC CTA T	NM_000402
TBP	Fw: CAA CAG CCT GCC ACC TTA CGC TC<br>Rev: AGG CTG TGG GGT CAG TCC AGT G	NM_003194
TUBA1A	Fw: GGC AGT GTT TGT AGA CTT GGA ACC C<br>Rev: TGT GAT AAG TTG CTC AGG GTG GAA G	NM_006009
ATF4	Fw: TGA TGT CCC CCT TCG ACC AGT C<br>Rev:TGT CGC TGG AGA ACC CAT GAG G	NM_182810
ATF6	Fw: CAG CGG AGC CAC TGA AGG AAG<br>Rev: TGA GTC TTG GGT GCT GCT GGA AG	NM_007348
CHOP	Fw: GTT AAA GAT GAG CGG GTG GCA GC<br>Rev: GGT GCT GCT TTC AGG TGT GGT GA	NM_001195054
HSPA5	Fw: CTGCGTCGGCGTGTTCAAGA<br>Rev: GCCAATCAGACGTTCCCCTTCA	NM_005347

ESM Table 2: Antibodies used in this study.

Protein	Supplier	Cat #	Dilution
JNK	Cell Signaling	9252	1:1000
p-JNK	Cell Signaling	4668	1:1000
p38	Cell Signaling	8690	1:1000
p-p38	Cell Signaling	9211	1:1000
ERK	Cell Signaling	4695	1:1000
p-ERK	Cell Signaling	9101	1:1000
IL1R1	R&D Systems	AF269	1:2000
IFNGR1	R&D Systems	MAB6731	1:500
TNFR1	Cell Signaling	3736	1:500

ESM Table 3: Top 30 of affected upregulated genes at different stages of differentiation.

Increased expression										
	hPSCs		DE		MPCs		SC-beta cells		EndoC-βH1	
No.	Gene	Fold Change [log2]	Gene	Fold Change [log2]	Gene	Fold Change [log2]	Gene	Fold Change [log2]	Gene	Fold Change [log2]
1	CH25H	1.48	APOL6	4.17	APOL6	4.87	BIRC3	5.85	APOL6	4.33
2	CXCL1	1.82	B2M	4.74	BIRC3	5.25	C3	6.58	BST2	5.65
3	CXCL5	1.15	BTN3A2	3.21	BTN3A2	4.74	CCL20	8.42	CXCL10	5.26
4	DNAH5	1.40	CXCL1	4.15	CCL2	5.35	CCL3	4.82	CXCL9	6.00
5	DRD4	1.05	CXCL2	4.32	CXCL11	5.77	CCL8	6.13	ETV7	5.21
6	EBI3	1.97	CXCL3	3.94	CXCL8	4.79	CHI3L1	5.36	GBP1	5.94
7	ERAP2	1.08	CXCL5	3.19	CXCL9	6.09	CXCL10	9.01	GBP3	4.86
8	IFIT2	1.21	DEFB4A	3.28	ERAP2	6.75	CXCL11	6.54	GBP5	4.92
9	IL32	2.23	EBI3	3.17	GBP1	5.58	CXCL2	4.99	GIMAP2	4.54
10	INHBA	1.87	ERAP2	5.07	GBP3	6.36	CXCL3	4.98	HCP5	5.26
11	IRF1	1.01	FAM150B	3.16	GBP5	6.09	CXCL5	5.66	HLA-B	5.14
12	LOC387720	1.53	GBP1	3.19	HCP5	6.11	CXCL6	4.93	HLA-F	5.49
13	LTB	5.60	HCP5	3.64	HLA-F	5.15	CXCL8	5.32	HLA-J	4.59
14	MIR146A	3.10	IFIH1	3.36	ICAM1	7.04	CXCL9	6.99	IFIT2	4.53
15	NNMT	1.09	IL15	3.16	IFIT2	4.97	DEFB4A	8.06	IFIT3	6.09
16	NPPB	1.28	IL32	5.01	IFIT3	5.55	DUOXA2	5.29	IRF1	4.45
17	RARRES3	1.00	IRF1	3.70	IL32	5.69	EBI3	6.39	LEUTX	4.80
18	SEMG1	1.27	KCNJ13	4.04	IRF1	5.23	ETV7	5.00	NLRC5	4.39
19	TMEM163	1.62	KCNJ16	3.38	MIR146A	5.56	GBP1	7.04	PARP14	4.83
20	TNF	2.54	LTB	4.69	NEURL3	4.74	GBP2	6.27	PATL2	4.32
21	UBD	3.33	MX1	3.36	PIGR	7.25	GBP5	6.22	PSMB9	5.59
22			NEURL3	3.16	PSMB8	6.46	ICAM1	5.29	RARRES3	6.13
23			PARP9	3.19	PSMB9	7.65	IDO1	4.94	RTP4	5.45
24			PSMB8	5.56	RARRES3	7.25	IL32	5.37	SAMD9L	4.75
25			PSMB9	4.82	RTP4	6.89	LCN2	6.20	SP110	4.88
26			RARRES3	3.31	SLC6A14	7.89	LTB	5.62	TAP1	5.10
27			TAP1	5.61	TAP1	6.51	PSMB9	6.10	UBD	5.33
28			TAP2	4.44	TAP2	5.21	REG3A	5.63	UBE2L6	4.42
29			TNFRSF9	3.50	TNF	6.14	TAP1	4.89	USP30-AS1	5.39
30			UBD	5.09	TNFRSF9	5.15	VNN2	5.72	XLOC_014422	4.51

ESM Table 4: Top 30 of affected downregulated genes at different stages of differentiation.

Decreased expression										
	hPSCs		DE		MPCs		SC-beta cells		EndoC-βH1	
No.	Gene	Fold Change [log2]	Gene	Fold Change [log2]	Gene	Fold Change [log2]	Gene	Fold Change [log2]	Gene	Fold Change [log2]
1	A_33_P3407049	-1.06	ADAMTS18	-2.30	A_33_P3258561	-3.33	A_33_P3389668	-2.25	A_33_P3229587	-1.37
2	BC040838	-1.58	ADAMTS8	-1.81	A_33_P3277679	-2.29	ATP1A2	-3.62	A_33_P3326151	-1.35
3	C17orf99	-1.33	ADRA2A	-2.69	AGR2	-3.19	COL8A1	-2.14	A_33_P3326942	-1.59
4	C6orf10	-1.21	ASIC1	-1.59	AGR3	-3.09	CORIN	-3.51	BNC2	-1.87
5	CFAP74	-1.23	BPIFA1	-1.45	AKR1C1	-2.39	CRABP1	-2.44	CATSPERB	-1.85
6	DNAH12	-1.26	CD34	-2.22	AQP2	-2.64	DPPA2	-2.98	CBLN2	-1.58
7	DRD5	-1.03	CLDN11	-1.91	BMP3	-3.04	ENST00000444823	-2.97	CYP2C19	-1.42
8	ENST00000476906	-1.52	FABP7	-1.58	C4orf51	-2.42	ENST00000590085	-3.70	CYR61	-1.30
9	ENST00000554907	-1.34	GALNT16	-1.48	CAPN8	-2.42	FAM159B	-2.24	EFCAB9	-1.78
10	FAT2	-1.33	GPM6A	-1.80	CD3G	-2.51	FAM181A	-2.82	ENST00000503220	-1.71
11	FLJ46066	-1.83	GRIK3	-2.17	CDRT15L2	-2.43	FOXN4	-2.83	EPHA5-AS1	-1.30
12	LILRB3	-1.26	GRP	-1.58	DKK2	-2.61	HHIP	-2.28	FAM149B1	-2.15
13	lnc-CNTN1-1	-1.18	GYPB	-1.51	EDN3	-3.43	ISLR	-2.47	FAM86DP	-1.73
14	lnc-SREK1-1	-1.60	GYPE	-1.59	EPHA7	-3.01	KCNIP3	-2.73	FILIP1L	-1.37
15	LOC100131043	-1.37	HDC	-1.54	FSHR	-2.55	KHK	-2.78	HS3ST4	-1.55
16	LOC283140	-1.18	LOC286189	-1.83	GHR	-2.37	LOC285740	-2.44	LINC00632	-1.85
17	LOC284581	-1.05	LOX	-1.60	GSTA2	-2.45	LOC90834	-2.99	LOC100130169	-1.79
18	LPA	-1.17	LRRTM1	-2.00	GSTA5	-2.45	LRTM1	-2.30	LOC100996890	-3.56

19	MUC5AC	-1.41	MEIS2	-1.49	KCNIP1	-2.69	MUSTN1	-2.49	LOC389332	-2.00
20	NT5DC4	-1.37	MIXL1	-1.51	LMO3	-2.41	NTN1	-2.59	LOC401410	-1.38
21	OR1J1	-1.02	PAX1	-2.39	NHS	-2.51	OGN	-3.84	NDST3	-1.73
22	PATL1	-1.44	PAX2	-1.91	PTPRT	-2.36	OMG	-2.67	NLRP11	-1.45
23	POM121L8P	-1.49	PDZRN4	-1.95	RAB27B	-2.28	PHOX2B	-2.25	P2RX7	-2.63
24	SEC14L3	-1.20	PIANP	-1.45	RAP1GAP	-2.39	PTF1A	-2.54	PAX9	-3.37
25	TMED9	-1.30	RAB3A	-1.53	RNA18S5	-3.47	SCARA5	-2.99	RASL11B	-1.32
26	TSPAN16	-2.06	SAMD3	-1.49	RNF222	-2.53	SCUBE1	-2.50	RBPJL	-2.26
27			SLC1A2	-1.70	SLC26A3	-3.46	TMEM100	-2.31	SOX8	-1.49
28			SLC7A14	-1.60	SLITRK6	-2.29	VWA5B1	-2.49	TEX26	-1.38
29			ST6GAL2	-1.48	TFF2	-2.30	WFDC1	-2.84	WDR12	-2.27
30			TENM4	-1.55	UNC13C	-2.67	WFIKKN2	-2.17	WEE2	-1.30

ESM Table 6: According to GO terms, ER stress and UPR-associated and in SC-beta cells and/or EndoC-βH1 cells regulated genes.

	SC-beta cells	EndoC-βH1		SC-beta cells	EndoC-βH1
HSPA5	0.19	1.07	***CREB3L3***	-1.4	●
CHOP	0.69	1.16	***CASP4***	3.05	2.53
THBS1	1.71	0	***CEBPB***	1.06	1.14
CCL2	4.8	●	***CFTR***	1.83	●
CXCL8	5.32	●	***NCCRP1***	1.7	0
PARP8	1.05	0.9	***PDIA4***	0.3	1.12
OPTN	0.37	1.37	***PML***	0.49	1.95
HSPB8	1.7	●	***RASGRF1***	-2	●
FBXO6	0.97	4.18			

(● = below threshold)

ESM Table 7: Comparative analysis of differential regulation of chemokines with data from human islets prepared by Eizirik et al. [4].

Chemokines						
Gene	Valid log2 Ratio (d00)	Valid log2 Ratio (d04)	Valid log2 Ratio (d14a+b)	Valid log2 Ratio (d28)	Valid log2 Ratio (Endoc)	Study Eizirik
CCL1	●	●	●	●	●	-
CCL2	0.22	1.25	5.35	4.80	●	Up
CCL3	●	●	●	4.82	●	Up
CCL4L2	●	●	2.42	3.38	●	-
CCL5	●	●	3.25	4.44	●	Up
CCL7	●	●	●	●	●	-
CCL8	●	●	●	6.13	●	Up
CCL11	●	●	●	●	●	-
CCL13	●	●	●	●	●	-
CCL14	●	●	●	●	●	-
CCL15	-0.34	●	-0.28	-0.28	0.11	-
CCL16	-0.01	-0.06	-0.02	-0.25	0.01	X
CCL17	●	●	●	●	●	-
CCL18	●	●	●	●	●	-
CCL19	●	●	-0.11	0.12	●	-
CCL20	●	●	2.56	8.42	●	Up
CCL21	●	●	●	3.38	●	-
CCL22	●	●	●	●	●	Up
CCL23	●	●	●	●	●	-
CCL24	-0.03	-0.15	-0.25	-0.30	-0.11	-
CCL25	0.06	2.30	●	●	●	-
CCL26	0.15	0.81	●	●	●	-
CCL27	-0.11	-0.08	0.12	-0.35	-0.35	-
CCL28	0.04	0.20	●	●	0.26	-
CXCL1	1.82	4.15	3.83	4.37	1.55	Up
CXCL2	0.83	4.32	3.62	4.99	1.18	Up
CXCL3	0.69	3.94	3.87	4.98	1.05	Up
CX3CL1	●	●	●	2.45	0.82	Up
PF4	0.23	●	●	0.63	-0.76	Up
CXCL5	1.15	3.19	3.59	5.66	0.11	Up
CXCL6	0.66	●	4.13	4.93	●	-
PPBP	●	●	●	●	●	-
CXCL8	●	●	4.79	5.32	●	X
CXCL9	●	●	6.09	6.99	8.68	Up
CXCL10	●	●	4.65	9.01	7.14	Up
CXCL11	●	●	5.77	6.54	2.08	Up
CXCL12	-0.03	0.10	1.32	0.25	0.09	-
CXCL13	●	●	●	●	●	-
CXCL14	0.39	0.39	-0.45	-0.56	1.67	-
CXCL16	-0.06	0.05	1.55	0.88	1.64	-
CXCL17	●	●	●	0.89	●	Up

XCL1	0.10	-0.90	0.28	0.00	●	-
FAM19A1	●	●	●	●	●	-
FAM19A2	0.26	-0.92	-1.52	0.08	-0.73	-
FAM19A3	●	●	●	●	●	-
FAM19A4	-0.17	●	●	-0.65	-0.54	-
FAM19A5	0.02	-0.62	-0.01	-0.09	-1.03	-

(X = not analyzed, ● = below threshold, - = not differentially expressed)

ESM Table 8: Comparative analysis of differential regulation of Cytokines with data from human islets prepared by Eizirik et al. [4].

Cytokines						
Gene	Valid log2 Ratio (d00)	Valid log2 Ratio (d04)	Valid log2 Ratio (d14a+b)	Valid log2 Ratio (d28)	Valid log2 Ratio (Endoc)	Study Eizirik
IL1A	●	●	●	●	●	Up
IL1B	●	●	●	3.51	●	Up
IL1RAP	-0.06	●	-0.68	-0.48	0.17	-
IL2	●	●	●	●	●	X
IL20	●	●	●	●	●	Up
IL22	●	●	●	●	●	-
IL25	●	●	●	●	●	X
IL27	●	●	●	●	0.56	-
IL26	●	●	●	●	●	-
IL24	●	●	●	●	●	Up
IL23A	0.25	0.16	3.84	2.72	0.40	Up
IL3	●	●	●	●	●	X
IL31	●	●	●	●	●	-
IL32	2.23	5.01	5.69	5.37	●	Up
IL33	●	●	●	2.50	●	Up
IL34	0.30	0.65	●	●	●	-
IL36A	●	●	●	●	●	X
IL36G	●	●	●	●	●	X
IL36RN	●	●	●	●	●	X
IL36B	●	●	●	●	●	X
IL37	0.09	0.20	●	●	-0.39	X
IL4	●	●	●	●	●	-
IL5	●	●	●	●	●	-
IL6	0.21	1.44	●	0.33	0.51	Up
IL7	●	●	●	1.09	1.22	Up
IL9	●	●	●	●	●	X
IL10	●	●	●	●	●	-
IL11	0.00	2.13	1.72	0.05	-0.38	Up
IL12A	●	●	●	●	●	-
IL12B	●	●	●	●	●	-
IL13	●	●	●	●	●	-
IL15	0.29	3.16	3.71	2.73	2.54	Up
IL16	-0.03	0.00	-0.57	-0.84	0.35	-

IL17A	•	•	•	•	•	-
IL17B	•	•	•	•	•	-
IL17C	0.36	•	•	•	•	Up
IL17D	0.06	-0.35	-0.31	-0.32	-0.71	-
IL17F	•	•	•	•	•	-
IL18	-0.07	•	•	•	•	Down
IL19	•	•	•	•	•	-
OSM	•	•	•	•	•	Up
CSF1	-0.03	0.29	0.35	1.42	1.75	Up
CSF2	•	•	•	•	•	Up
CSF3	•	•	•	•	•	Up
TNF	2.54	•	6.14	•	2.09	Up
TNFSF13	•	•	•	1.48	•	Up
TNFSF9	0.22	0.58	0.41	1.24	•	-
CD27	•	•	•	•	•	-
TNFSF8	•	•	•	•	•	-
FASLG	•	•	•	•	•	-
TNFSF18	•	•	•	•	•	-
TNFSF4	0.18	0.30	-0.41	-0.21	•	-
TNFSF13B	•	0.20	•	0.31	-0.05	Up
TNFSF10	•	•	4.29	0.60	1.65	-
TNFRSF12A	0.01	0.90	1.11	1.09	0.31	Up
TNFSF11	0.20	•	•	•	0.34	-
IFNG	•	•	•	•	•	-
IFNA2	•	•	•	•	•	X
IFNW1	•	•	•	•	•	-
IFNE	•	0.07	•	•	•	-
IFNB1	•	•	•	•	•	-
IFNK	•	•	•	•	•	-
IFNL1	•	•	•	•	•	X
IFNL2	0.21	0.02	•	•	•	X
IFNA7	•	•	•	•	•	-
IFNA8	•	•	•	•	•	-
IFNA4	-0.42	0.30	•	•	0.26	-
IFNA14	•	•	•	•	•	-
IFNA10	•	•	•	•	•	-
IFNA16	•	•	•	•	•	X
IFNA6	•	•	•	•	•	-
MST1	-0.10	-0.69	-0.66	-0.61	0.06	Down
KITLG	0.08	-0.31	0.47	0.83	-0.78	Down
THPO	•	•	•	•	•	-
EPO	0.00	•	•	•	•	-
LIF	0.04	0.50	2.00	1.96	•	Up
TGFA	-0.02	0.53	0.76	0.33	1.00	-
TGFB1	-0.22	-0.28	-0.06	-0.01	•	-
TGFB2	•	2.04	0.21	0.41	•	Down
TGFB3	•	-0.95	-0.66	0.59	-0.59	Down
CD40LG	•	•	•	•	•	X

(X = not analyzed, • = below threshold, - = not differentially expressed)

ESM Table 9: Comparative analysis of differential regulation of the GBP-family with data from human islets prepared by Eizirik et al. [4].

GBP family						
**Gene**	**Valid log2 Ratio (d00)**	**Valid log2 Ratio (d04)**	**Valid log2 Ratio (d14a+b)**	**Valid log2 Ratio (d28)**	**Valid log2 Ratio (Endoc)**	**Study Eizirik**
GBP1	●	3.19	5.58	7.04	8.65	Up
GBP2	●	2.55	3.40	6.27	2.71	Up
GBP3	●	●	6.36	4.38	6.09	Up
GBP4	0.08	0.97	2.50	4.18	4.45	Up
GBP5	●	●	6.09	6.22	6.14	Up
GBP6	0.22	0.36	-0.58	-0.14	0.13	-

(● = below threshold, - = not differentially expressed)

ESM Table 10: Comparative analysis of differential regulation of MHC class I-genes with data from human islets prepared by Eizirik et al. [4].

MHC class genes						
**Gene**	**Valid log2 Ratio (d00)**	**Valid log2 Ratio (d04)**	**Valid log2 Ratio (d14a+b)**	**Valid log2 Ratio (d28)**	**Valid log2 Ratio (Endoc)**	**Study Eizirik**
HLA-A	0.05	1.41	2.60	1.71	3.28	-
HLA-B	0.17	1.40	4.01	2.14	6.58	-
HLA-C	0.10	1.02	1.32	0.92	3.93	Up
HLA-E	0.14	1.40	2.53	1.86	3.94	Up
HLA-F	0.27	2.53	5.15	4.13	7.99	-
HLA-G	0.06	1.33	2.26	1.53	3.69	-
TAP1	0.58	5.61	6.51	4.89	6.28	Up
TAP2	0.28	4.44	5.21	3.64	4.53	Up
TAPBP	0.36	2.68	3.23	1.53	2.32	Up
ERAP1	0.31	0.82	2.29	0.89	1.50	Up
IDE	-0.02	0.07	0.22	0.36	0.15	-
ERAP2	1.08	5.07	6.75	3.67	3.78	-
B2M	0.69	4.74	3.18	1.69	2.37	-
PSMB9	0.80	4.82	7.65	6.10	8.31	Up

(- = not differentially expressed)

ESM Table 11: Comparative analysis of differential regulation of pattern recognition receptors (PRRs) with data from human islets prepared by Eizirik et al. [4].

Pattern Recognition Receptors						
**Gene**	**Valid log2 Ratio (d00)**	**Valid log2 Ratio (d04)**	**Valid log2 Ratio (d14a+b)**	**Valid log2 Ratio (d28)**	**Valid log2 Ratio (Endoc)**	**Study Eizirik**
TLR1	●	●	●	0.67	0.88	-
TLR2	0.20	0.46	2.27	2.47	●	-
TLR3	0.05	●	●	●	●	Up
TLR4	●	●	0.75	1.48	●	-
TLR5	-0.04	-0.06	0.40	0.08	-0.07	-
TLR6	●	●	●	●	●	-
TLR7	●	●	●	●	●	-
TLR8	●	●	●	●	●	-
TLR9	●	●	●	●	●	-
TLR10	●	●	●	-1.04	●	-
NOD1	0.13	0.04	0.18	0.14	0.21	-
NOD2	-0.07	●	●	●	●	Up
NLRC3	-0.19	0.69	-0.78	-0.25	0.17	-
NLRC4	●	●	●	●	●	-
NLRC5	0.28	2.06	2.16	3.01	5.26	Up
NLRP1	●	●	●	●	●	-
NLRP2	0.04	-0.02	0.00	0.11	●	-
NLRP3	0.07	●	●	●	●	-
NLRP4	●	●	●	●	●	-
NLRP5	●	●	●	●	●	-
NLRP6	●	●	●	●	●	-
NLRP7	0.75	1.63	●	●	●	-
NLRP8	●	●	●	●	●	-
NLRP9	●	●	●	●	●	-
NLRP10	●	●	●	●	●	-
NLRP11	●	●	●	●	-1.45	-
NLRP12	0.03	0.39	●	●	●	-
NLRP13	●	●	●	●	●	-
NLRP14	●	●	●	●	●	-
DDX58	0.33	0.14	1.08	1.08	0.93	-
IFIH1	0.93	3.36	4.52	0.64	1.89	-
DHX58	-0.03	0.58	1.00	1.23	2.12	-
CIITA	●	●	●	●	●	Up
NAIP	-0.29	-0.03	0.03	-0.35	-0.18	-

(- = not differentially expressed)

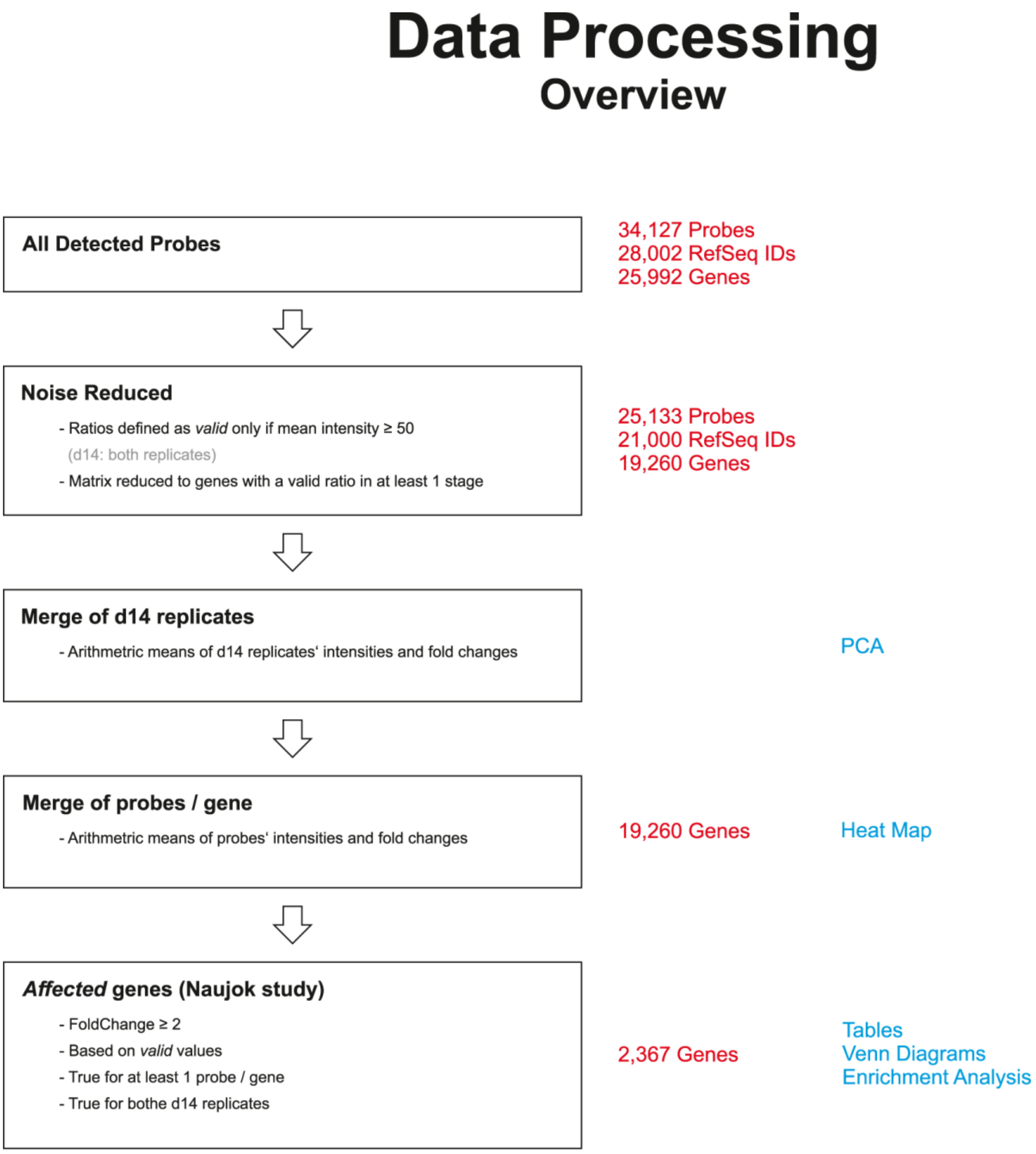

ESM Fig. 1. Workflow of of data processing using the Perseus software.

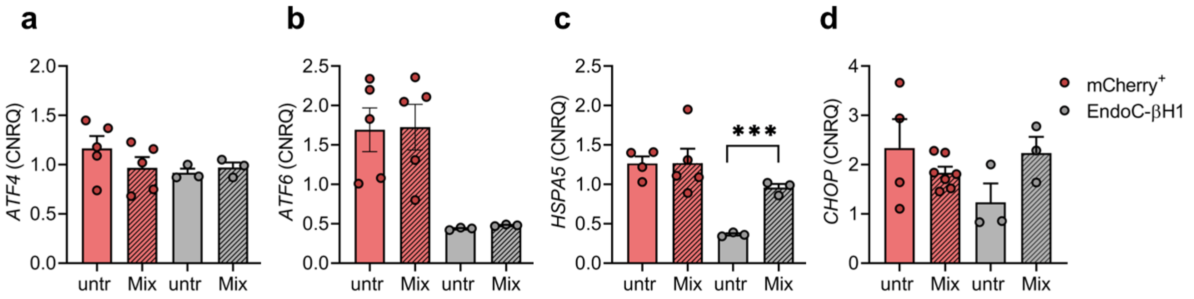

ESM Fig. 2. RT-qPCR-analysis of ER-stress marker genes.

RT-qPCR gene expression analysis of *ATF4* **(a)**, *ATF6* **(b)**, *HSPA5* **(c)** and *CHOP* in 24 h cytokine-treated (Mix) compared to untreated (untr) SC-beta cells (mCherry^{+}) and EndoC-βH1 cells. Values are means ± SEM. *** =p <0.001, n=3-7. Two-tailed, unpaired *Student's* t-test. Data are represented as CNRQ, calibrated normalised relative quantity.

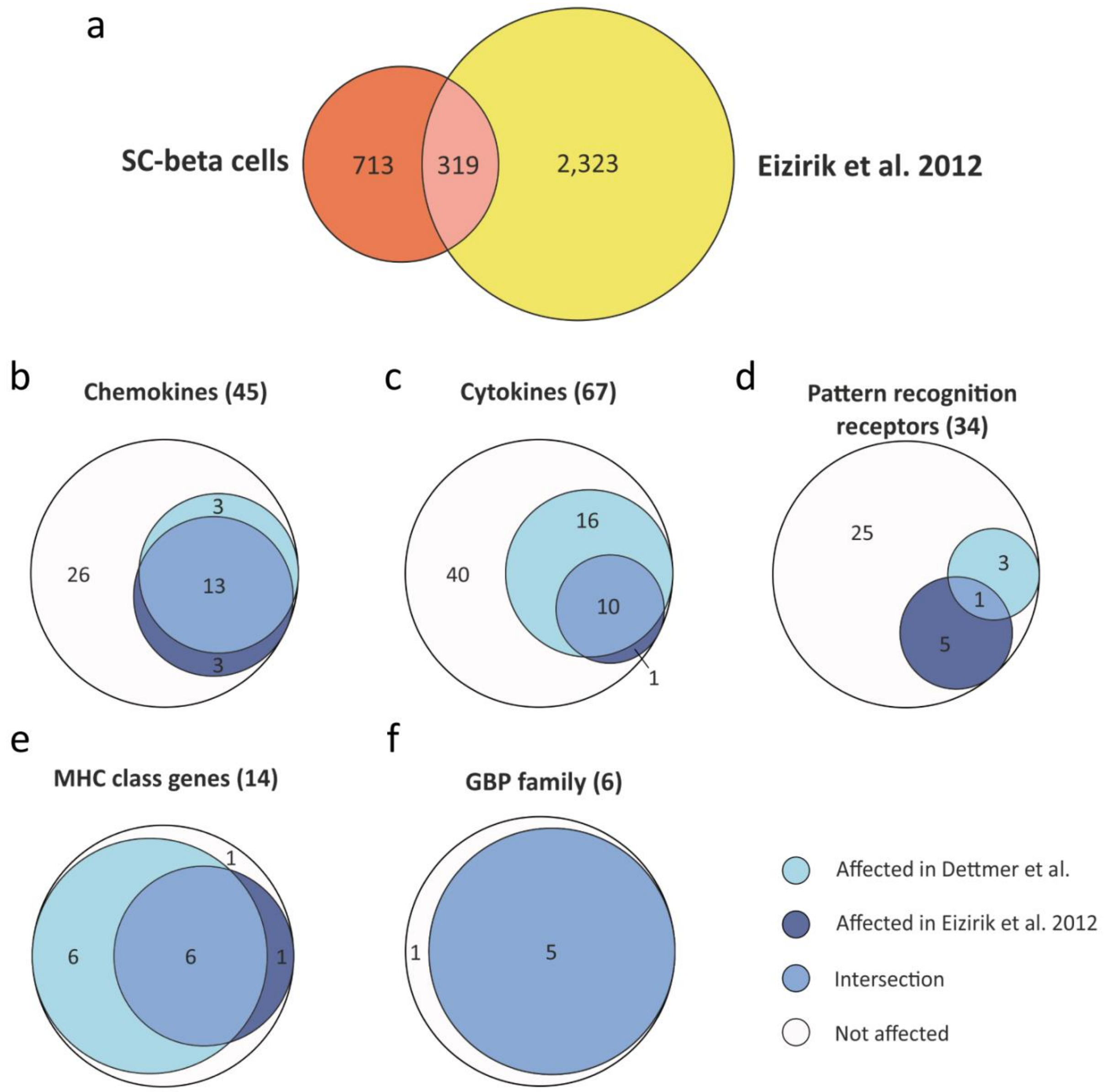

ESM Fig. 3. Comparison of studies SC-beta cells vs human islets (Eizirik et al. [4]). (a) Venn diagrams showing the number of affected genes in and the overlap between both studies. **(b-f)** Numbers of genes affected (up-and downregulated) by cytokine treatment in SC-beta cells or human islets annotated as chemokines **(b)**, cytokines **(c)**, pattern recognition receptors (PRR) **(d)**, MHC class I genes **(e)** and the interferon-induced guanylate-binding protein (GBP) family **(f)**.

ESM Literature

[1] Dettmer R, Niwolik I, Mehmeti I, Jörns A, Naujok O (2021) New hPSC SOX9 and INS Reporter Cell Lines Facilitate the Observation and Optimization of Differentiation into Insulin-Producing Cells. Stem cell reviews and reports. 10.1007/s12015-021-10232-9

[2] Grunnet LG, Aikin R, Tonnesen MF, et al. (2009) Proinflammatory Cytokines Activate the Intrinsic Apoptotic Pathway in β-Cells. Diabetes 58(8): 1807-1815. 10.2337/db08-0178

[3] Eizirik DL, Mandrup-Poulsen T (2001) A choice of death--the signal-transduction of immune-mediated beta-cell apoptosis. Diabetologia 44(12): 2115-2133. 10.1007/s001250100021
[4] Eizirik DL, Sammeth M, Bouckenooghe T, et al. (2012) The human pancreatic islet transcriptome: expression of candidate genes for type 1 diabetes and the impact of pro-inflammatory cytokines. PLoS genetics 8(3): e1002552. 10.1371/journal.pgen.1002552
